# Looking for Local Adaptation: Convergent Microevolution in Aleppo Pine (*Pinus halepensis*)

**DOI:** 10.3390/genes10090673

**Published:** 2019-09-04

**Authors:** Rose Ruiz Daniels, Richard S. Taylor, Santiago C. González-Martínez, Giovanni G. Vendramin, Bruno Fady, Sylvie Oddou-Muratorio, Andrea Piotti, Guillaume Simioni, Delphine Grivet, Mark A. Beaumont

**Affiliations:** 1 Department of Forest Ecology and Genetics, Forest Research Centre, INIA, Carretera A Coruña km 7.5, 28040 Madrid, Spain; 2 The Roslin Institute, The University of Edinburgh, Easter Bush Campus, Midlothian, EH25 9RG Scotland, UK; 3 School of Earth Science, University of Bristol, Bristol Life Sciences Building 24 Tyndall Avenue, Bristol BS8 1TQ, UK; 4 BIOGECO, INRA, Univ. Bordeaux, 33610 Cestas, France; 5 Institute of Biosciences and Bioresources, National Research Council, I-50019 Sesto Fiorentino, Florence, Italy (G.G.V.) (A.P.); 6 INRA, URFM, Ecologie des Forêts Méditerranéennes, 84914 Avignon, France (B.F.) (S.O.-M.) (G.S.)

**Keywords:** *Pinus halepensis*, natural selection, outliers, SNP, altitudinal gradients

## Abstract

Finding outlier loci underlying local adaptation is challenging and is best approached by suitable sampling design and rigorous method selection. In this study, we aimed to detect outlier loci (single nucleotide polymorphisms, SNPs) at the local scale by using Aleppo pine (*Pinus halepensis*), a drought resistant conifer that has colonized many habitats in the Mediterranean Basin, as the model species. We used a nested sampling approach that considered replicated altitudinal gradients for three contrasting sites. We genotyped samples at 294 SNPs located in genomic regions selected to maximize outlier detection. We then applied three different statistical methodologies—Two Bayesian outlier methods and one latent factor principal component method—To identify outlier loci. No SNP was an outlier for all three methods, while eight SNPs were detected by at least two methods and 17 were detected only by one method. From the intersection of outlier SNPs, only one presented an allelic frequency pattern associated with the elevational gradient across the three sites. In a context of multiple populations under similar selective pressures, our results underline the need for careful examination of outliers detected in genomic scans before considering them as candidates for convergent adaptation.

## 1. Introduction

Finding the genetic basis of local adaptation is a topic of crucial importance in evolutionary biology as it allows for the study of the mechanisms of natural selection [1,2]. However, there is still no consensus on how widespread local adaptation is as well as under which conditions it occurs. Studies based on reciprocal transplant and common garden experiments showed that plants can be locally adapted [3,4] and that local adaptation is more common for large populations. However, generalizing this phenomenon depends on the definition of local adaptation (relaxed versus a strict definition, [3]). Forest trees provide numerous examples of adaptive genetic divergence [5] as they generally combine a wide distribution across contrasting environmental conditions, large population sizes, and extensive levels of gene flow that may increase the rate of adaptation as well as high levels of genetic variation for fitness-related traits. However, examples that show that forest trees can genetically adapt at small geographical scales are limited [6]. The few studies that have looked at local adaptation in trees contemplating adaptive molecular divergence (e.g., [7,8,9,10]) or correlations between adaptive genetic variants and environmental variables [11,12,13] illustrate that the existence of spatially heterogeneous selective pressure may favor locally specialized genotypes despite extensive gene flow at the local scale.

In the present study, our objective was to shed new light on the genomic signatures of local adaptation in Aleppo pine (*Pinus halepensis*, Mill.). Aleppo pine has a circum-Mediterranean distribution that is both geographically fragmented and vast, spanning 3.5 million hectares [14]. This very drought resistant species is able to colonize and adapt to many habitats around the Mediterranean Basin as well as become invasive in southern hemisphere Mediterranean habitats [15,16]. Its environmental versatility lies both in its high plasticity [17,18] as well as, potentially, in its genetic adaptation to the Mediterranean region [19,20]. In Europe, paleoecological and fossil records as well as genetic data suggest that the Aleppo pine demographic history is characterized by different colonization and drift events, resulting in its genetic diversity following a longitudinal trend as many other species in the Mediterranean Basin [20,21]. This left Aleppo pine with a complex genetic pattern made of two main genetic clusters: an eastern/southern cluster that is more genetically diverse than the western cluster [20,22,23,24], with both being connected by population admixture [20,25]. The evolutionary history of Aleppo pine, combined with its ecological versatility in many Mediterranean habitats, constitute a good setting to test whether patterns of genetic divergence among heterogeneous environments hold across phylogeographic groups.

Ruiz Daniels et al. [20] previously investigated selection imprint in 44 populations of Aleppo pine covering the species range. Here, we focused on seven populations sampled along climatic gradients and aimed to detect convergent adaptation by combining the following. (1) A nested design of natural populations from distinct phylogeographic groups and contrasted environments, where we selected replicated populations in each site across two genetic clusters. Replicated populations were sampled at distinct elevations to reflect a combination of environments including differences in precipitation and temperature, two climatic variables known to affect the distribution and adaptation of this conifer [20,25,26,27,28,29]. Several studies have shown morphological and physiological adjustments of woody species along elevational gradients [30,31], which seem to therefore constitute relevant selection drivers. (2) A set of single nucleotide polymorphisms (SNPs) located in genomic regions selected to maximize outlier detection (see more details in the material and method section). The 294 SNPs genotyped in the seven populations fall within loci that were found to be under selection in pine species including Aleppo pine [19,20,24,32]. (3) A combination of methods that look at outlier loci based on environmental correlations or genetic differentiation. We took advantage of three recently developed statistical methods that correct for population structure: PCAdapt [33], Bayenv2 [34,35], and Baypass [36]. This attribute allows these methods to outperform methods that do not [37,38,39,40] and is a critical issue when there is evidence of shared history and gene flow among populations, as in Aleppo pine. The originality of the present work lies on the combination of these approaches to maximize the detection of outlier SNPs involved in convergent evolution in Aleppo pine.

## 2. Materials and Methods 

### 2.1. Sampling 

#### 2.1.1. Plant Material and Climatic Data

**Figure 1:**
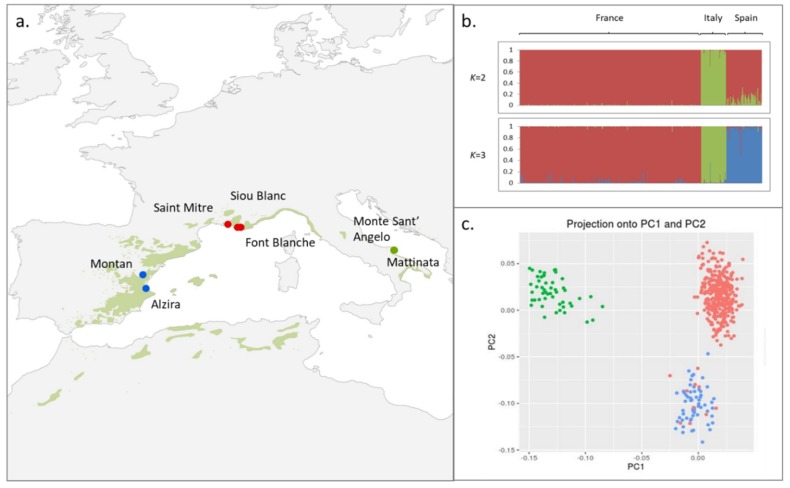
Aleppo pine population structure. **(a)** Localization of the three populations superimposed on the natural range of Aleppo pine from EUFORGEN (green area). **(b)** Bayesian clustering performed in STRUCTURE for the seven populations. Population order from left to right is as follows: France (Font Blanche, Siou Blanc, Saint Mitre), Italy (Monte Sant’ Angelo, Mattinata), and Spain (Montan, Alzira). **(c)** Score plot of SNP data where each country is color coded (France: red, Italy: green, and Spain: blue). This plot displays the projections of the individuals onto the first PC (PC1) and the second PC (PC2).

Adult trees from three sites in three different countries spanning the two main phylogeographic clusters of Aleppo pine were sampled from population pairs or triplets: three French populations (356 individuals), two Italian populations (50 individuals), and two Spanish populations (70 individuals), totaling 476 individuals from seven populations (Figure 1a; see Tables S1 and S2 for more details). The sites are characterized by contrasted climatic conditions along altitudinal gradients. The aim of this sampling design was to maximize the differentiation between environments, while minimizing the differences in evolutionary history (gene flow should reduce differentiation at loci not under selection) [2]. This approach has been shown to be effective in comparative studies using simulated data [39] as well as when inferring outlier loci in empirical studies [12].

For each population, the 19 bioclimatic variables for the period 1950–2000 (available from WorldClim [41]) were downloaded to explore SNP-environment associations (see Table S3). The accuracy of this coarse grain climatic dataset was checked with local climatic datasets for the climatic variables based on precipitation and temperature. Specifically, WorldClim variables for precipitation and temperature were compared with those from a functional phytoclimatic model based on raw data from meteorological stations [42] for the Spanish populations, and from local meteorological stations for the French populations. Furthermore, elevation was added as a potential driver of selection as it encompasses a combination of geophysical influences [43], bringing the total of environmental variables to 20 (see Table S2 for more details).

#### 2.1.2. DNA Extraction, SNP Genotyping, and Gene Annotation

For every genotyped tree, 50 mg of needles were dried with silica gel and ground in a QIAwell (Qiagen, Venlo, the Netherlands) plate homogenized with a mixer mill MM300 (RETSH, Haan, Germany) under liquid nitrogen. DNA extractions were carried out with the Invisorb DNA plant HTS 96 kit (Invitek, Hayward, CA, USA) following the manufacturer’s instructions. DNA was quantified with Nanodrop 10,000 (Thermo Fisher Scientific, Wilmington, DE, USA).

Aleppo pine populations were successfully genotyped at 294 SNPs (conversion rate of 76.56%) using a 384-plex SNP assay with Illumina VeraCode technology as described in Pinosio et al. [44]. SNP genotypes are available at [45]. These SNPs originated from two sources. (i) 60% were identified from transcriptomic data coming from two Aleppo pine individuals with contrasting fire-response phenotypes [44]. A subset of these SNPs may be involved in adaption to fire as shown by the genetic association of some SNPs with serotiny [46]; and (ii) 40% were obtained by resequencing some loci originally developed in loblolly pine [47] as well as candidate genes from Grivet et al. [19,24]. Some of the loci involved in abiotic stress responses (e.g., cold, heat, drought, oxidative stresses) and phenology/photosystems have been shown to be under selection in pines, including that of Aleppo pine [19,20,24,32].

Loci for which SNPs were found putatively under selection using at least two different methods were annotated from homology with other plant species using Geneious version 6.1 [48] by searching against known loblolly pine EST contigs and the National Center for Biotechnology Information reference plant protein database.

### 2.2. Statistical Analysis

We used two Bayesian outlier approaches, Bayenv2 [34,35] and Baypass [36], as well as one method based on principal component analysis (PCA), implemented in the software PCAdapt [33]. Since methods for detecting outlier loci are based on underlying hypotheses that take into account different signals left by natural selection at the molecular level, there are inevitable discrepancies in the set of outlier loci [2,33,35,36,49]. Deciding which loci will be retained as candidates for further investigation is challenging, as is demonstrating that these outliers are candidates for convergent adaptation when detected across parallel elevational gradients.

#### 2.2.1. Population Structure 

To confirm that the population pairs/triplets were sampled from the same phylogeographic clusters, population genetic structure was estimated in two ways. First, pairwise *F*_ST_ was computed among all seven populations with Genepop v4.1 [50]. Second, the population genetic structure was inferred using the Bayesian clustering method STRUCTURE [51], with the following parameters: number of clusters (*K*) set from 1 to 10; number of iterations per *K* set to 10; number of steps set to 100,000 with a burn in period of 10,000 to minimize the effect of the starting configuration, and with an ancestry model of admixture. The convergence toward reliable allele frequency estimates in each genetic group and membership probabilities of individuals to a genetic group was assessed by checking that the alpha parameter was relatively constant, as indicated in the STRUCTURE manual.

#### 2.2.2. Looking for Outliers Using PCA

The rationale of this method is that the *F*_ST_ index of genetic variation can also be viewed as the proportion of variance explained by principal components [52,53,54,55,56]. First, the number of principal components to be retained (*K*) was established by running the PCA analysis in PCAdapt [33] with a large enough number of components (10 in this case). The *K* displaying most of the cumulative explained variance was chosen based on a scree plot, which presents the percentage of variance explained by each principal component (PC) in decreasing order. The choice of *K* was also corroborated by plotting individuals on the first two components (called a score plot by PCAdapt) to verify if the level of clustering was consistent with the chosen value for *K*. To account for missing data, the correlation matrix between individuals was computed using only the markers available for each individual. The Mahalanobis distance was used to identify the outliers by measuring how distant a data point is from the multivariate space’s centroid (overall mean), considering the covariance structure of all of the data points in the sample. By default, alleles displaying MAF < 5% were removed in the PCAdapt analyses.

Both Manhattan and Quantile—Quantile plots were used to visualize the distribution of the data. The presence of outliers was confirmed by applying a false discovery rate (FDR), defined as the percentage of false positives among the list of candidate SNPs. The FDR was set at 10%, as recommended by Luu et al. [33] using the R package qvalue (R Core Team 2013) to compute the *q*-values based on the *p*-value distribution.

#### 2.2.3. Looking for Outliers Using Bayesian Approaches

Bayesian approaches have been shown to perform well in situations where there is a non-uniform population structure [37,57]. The Baypass method is based on a very similar model as the Bayenv, with some improvements that allow for the better identification of significant outliers (see below). The two methodologies were used together with the intention of using the significance threshold for the *XtX* in Baypass to help define a cut-off point for the Bayenv outputs, for which standardized methods exist neither for the *XtX* statistics nor for the Bayes factors. The comparison aimed to also compare the outliers identified by the two methods.

- Bayenv

The method described in Coop et al. [34] and implemented in the Bayenv 2.0 package [35] estimates the population differentiation statistic *XtX* and evaluates the association of ecological variables with genetic marker differentiation. Bayenv2 first estimates the empirical pattern of covariance in allele frequencies between populations, and uses this as the null model to test each individual SNP for selection. To minimize the stochasticity in estimating the null model, three covariance matrices were produced from 100,000 iterations each, and the mean of these matrices (hereafter called mean covariance matrix) was then used. In order to test whether the mean covariance matrix represented the true variance of allele frequencies well across populations, it was compared to a pairwise *F*_ST_ matrix using a Mantel test in R (R Core Team 2013) with 1000 permutations. 

This covariance matrix, which takes into account correlations due to population structure, was then used as the null model in two analyses aimed at detecting SNPs under selection. (1) Detecting outliers through differences in overall genetic differentiation as in classic outlier tests by estimating the *XtX* averages across multiple samples from the MCMC. Outlier *XtX* values from the main *XtX* distribution were considered as potentially under selection. Finally, these values were compared to the ones estimated using Baypass in order to ascertain significance. Indeed, in theory, Baypass and Bayenv2 both produce *XtX* statistics and can be easily compared against each other. However, Bayenv2 does suffer from a lack of pre-defined methods to establish outliers based on *XtX* statistics, which would have been difficult to do without the results from Baypass to use as a guideline. (2) Finding SNPs that show a significant correlation between an environmental variable and allele frequencies. This was carried out by comparing, via Bayes factors, a model that allows for a linear relationship between allele frequency from the 294 SNPs and the 20 environmental variables with a null model based on the covariance matrix. This approach was repeated three times for each combination of SNP-environmental variable tested for association to account for instability between independent runs [38]. The mean of the three runs was then used to infer the final Bayes factors (BF). The Kass and Raftery [58] criterion was then used to determine the probability of these SNPs under selection, with 2lnK values above six (BF > 20) classified as strong.

- Baypass

The Baypass method is based on a very similar model as Bayenv2, but is suggested to be an improvement in two aspects: (i) It has a higher accuracy in the estimation of the baseline (ancestral) allele frequency through the use of a hierarchical Bayesian model; and (ii) the use of simulated pseudo observed datasets (PODs) from the posterior predictive distribution in order to calibrate the *XtX* statistics and determine a cut-off point for detecting potentially significant outliers by applying a false discovery rate threshold.

Baypass [36] was run using the core model, and R (R Core Team 2013) was used to analyze and visualize outputs. First, the correlation matrix $\hat{\Omega }$ was computed and estimates of *XtX* differentiation were produced from the SNP data. The correlation matrix was then visualized as a hierarchical cluster tree in order to be compared to a neighbor joining tree of pairwise *F*_ST_ done in Genepop v4.1 [50]. Genetic distances between populations were computed using the R package APE [59]. Second, a POD was constructed using the simulate_baypass function in R with 1000 SNPs by estimating the posterior mean of the hyperparameters a_pi and b_pi, which specify the beta priors for Pi, the baseline (ancestral) frequency at each locus, and then simulating samples with these parameter values. The POD was then analyzed in the same way as the real data to produce values of *XtX*. The quantiles of these empirical *XtX* distributions were used to calibrate the *XtX* observed for each locus in the original data, and the 99% quantile of the *XtX* distribution from the POD analysis provided a 1% threshold *XtX* value (the default cut-off in Baypass). This threshold was then used to discriminate between outlier and neutral SNPs. Baypass is expected to outperform Bayenv2 because it uses the analyzed data to provide hyperparameters for the outlier detectors. However, as the two methods have not been used in conjunction, we ran them concomitantly to compare how these different features will impact outlier detection.

Baypass was also used to detect outlier SNPs showing a significant correlation between an environmental variable and allele frequencies. This was carried out by comparing a model that allows for a linear relationship between allele frequency and environmental variable with a null model using only the correlation matrix. For this purpose, Baypass was run using an importance sampling estimator (IS) model based on Coop et al. [34]. The empirical Bayesian p-value (eBPis) was calculated for each SNP-environmental relationship, allowing for evaluation of the support in favor of a non-null regression coefficient when the eBPis is above three [36]. As there is high variability among runs when using this feature in Bayenv2, it is advisable to run multiple analyses and then average the Bayes factors [38]. This is not the case with Baypass, where no such variability exists.

In the two Bayesian analyses, SNPs with a minimum allele frequency (MAF) below 5% were kept (contrary to the PCA-based method, where by default these SNPs are removed) as their removal is not recommended because some of these might be under selection.

## 3. Results

### 3.1. Population Structure

Pairwise population *F*_ST_ were all significant except between the two Italian populations (Table S4). Moreover, populations within each country were the least differentiated, while among countries, the French and Spanish populations were genetically closer to each other than to the Italian populations (Table S4). This result is in line with the Bayesian clustering that revealed an optimal grouping for *K* = 2 and *K* = 3 (Figure 1b and Figure S1), and as for *K* > 3, the structure signal was lost (data not shown) and the variance across the iterations increased (Figure S1). This grouping is in full agreement with the PCA analysis run with PCAdapt where the main proportion of explained variance lies mainly in two PCs; more specifically, PC1 separates the Italian populations from the French and Spanish populations (this scenario corresponds to *K* = 2 with STRUCTURE), while PC2 separates the French populations from the other two to a lesser degree (Figure 1c). The genetic closeness between the French and Spanish populations was also reflected by admixed individuals between the two countries as shown in Figure 1b,c. Furthermore, they shared other characteristics such as lower genetic diversity when compared to the Italian ones.

### 3.2. Looking for Outliers Using PCA

The scree plot clearly showed that most of the variation was accounted for at *K* = 3 (with the main proportion of explained variance for the two first PCs), and that it was unnecessary to use more PCs (Figure S2), as further confirmed by inspecting the scatterplot of the first two PCs (Figure 1c). The distribution of the *p*-values was visualized with a Manhattan plot and a QQ-plot, and then used to compute the *q*-values (Figure S3). Eleven outliers were detected (Table 1), setting *q*-values at a false discovery rate of 10%.

### 3.3. Looking for Outliers Using Bayesian Approaches

- Bayenv

Confidence in the mean covariance matrix representing the true variance of allele frequencies across populations was assessed by the similarity between its heat map and that of pairwise *F*_ST_ values (Figure S4), that was found to be statistically significant according to the Mantel test (R^2^ = 0.801; *p*-value = 0.009).

The four top outliers from the *XtX* analysis were: SNP 4 (seq-9882-801), SNP 149 (seq-8671-529), SNP 316 (seq-10373-2483), and SNP 378 (seq-2_3941_01-381) (Table 1; Figure S5, left). When the Bayesian linear model was used with the 20 different environmental variables, four potential outlier SNPs were detected, classified as strong candidates for selection (20 < BF < 150) according to the Kass and Raftery (1995) criterion: SNP 169 (seq-0_10162_01-244), SNP 312 (seq-UMN_3408_01-293), SNP 316 (seq-10373-2483), and SNP 378 (seq-2_3941_01-381). Six environmental variables were involved in these correlations: elevation, BIO 2 (mean diurnal range), BIO 9 (mean temperature of driest quarter), BIO 12 (annual precipitation), BIO 16 (precipitation of wettest quarter), and BIO 19 (precipitation of coldest quarter) (Table S5).

- Baypass

Confidence in the correlation matrix $\hat{\Omega }$, was first assessed by visually comparing the $\hat{\Omega }$ values among the populations (Figure S6). The correlation matrix can be viewed as a hierarchical cluster tree (Figure S7, left), where the relationships of the seven populations were similar to those found with a neighbor joining tree based on pairwise *F*_ST_ (Figure S7, right), with populations from the same country clustering together (the exception being the French population from Font Blanche in the hierarchical cluster based on $\hat{\Omega }$ that includes much more individuals, from five to ten times more than the other studied populations). Two outlier SNPs were observed: SNP 149 (seq-8671-529) and SNP 378 (seq-2_3941_01-381) (Table 1; Figure S5, right).

When Baypass was used to detect SNPs showing significant correlation with environmental variables based on eBPis > 3, 17 SNPs were detected (see Table S6). These were then compared to the ones in Bayenv2 and only the outliers detected using both methods for the same environmental variable were included as strong candidates for selection, leading to a total of three SNPs: SNP 169 (seq-0_10162_01-244), SNP 316 (seq-10373-2483), and SNP 378 (seq-2_3941_01-381) (Table 2).

### 3.4. Combined Results of All Outlier Tests

**Figure 2:**
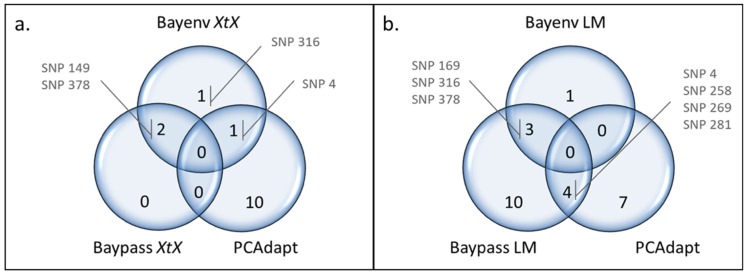
Venn diagrams comparing the outliers detected using PCAdapt with those identified using **(a)** the *XtX* statistics from Bayenv2 and Baypass, and **(b)** the Bayesian linear model (LM) from Bayenv2 and Baypass. Only the SNPs found under selection in more than one method are indicated.

Eight SNPs were identified as outliers in at least two methods (Figure 2): SNP 4, SNP 149, SNP 169, SNP 258, SNP 269, SNP 281, SNP 316, and SNP 378.

These SNPs belonged to six different loci, of which two could be annotated using the BLAST search (Table 3): seq-9882 (SNP 4 and SNP 258) corresponds to a PIN-like protein, while seq-10373-2483 (SNP 316) codes for a putative alpha-xylosidase (XYL1).

Two outliers were detected with both Bayenv2 and Baypass making use of *XtX* statistics (SNP 149 and SNP 378), while Bayenv2 detected two additional ones (SNP 4 and SNP 316). Of the eleven outliers detected by PCAdapt, only SNP 4 was in common with the *XtX* outliers found in Bayenv2 (Figure 2a). Bayenv2 and Baypass revealed three outlier SNPs correlated with the same environmental variables (Figure 2b): SNP 169 was found in association with BIO9 (mean temperature driest quarter), SNP 316 with elevation, and SNP 378 with BIO12 (annual precipitation). The *XtX* and environmental association approaches revealed two outliers in common in Bayenv (SNP 316 and SNP 378; Table 1 and Table S5) and one in Baypass (SNP 378; Table 1 and Table S6). Out of the 11 SNPs detected with PCAdapt, four were in common with those detected by Baypass using environmental correlations (SNP 4, SNP 258, SNP 269, and SNP 281; Figure 2b), all associated with BIO12 (annual precipitation) (Table 4). Finally, SNP 316 detected with Bayenv2 based on the *XtX* statistics was also detected in environmental correlations with Bayenv2 and Baypass (Figure 2a,b).

**Figure 3:**
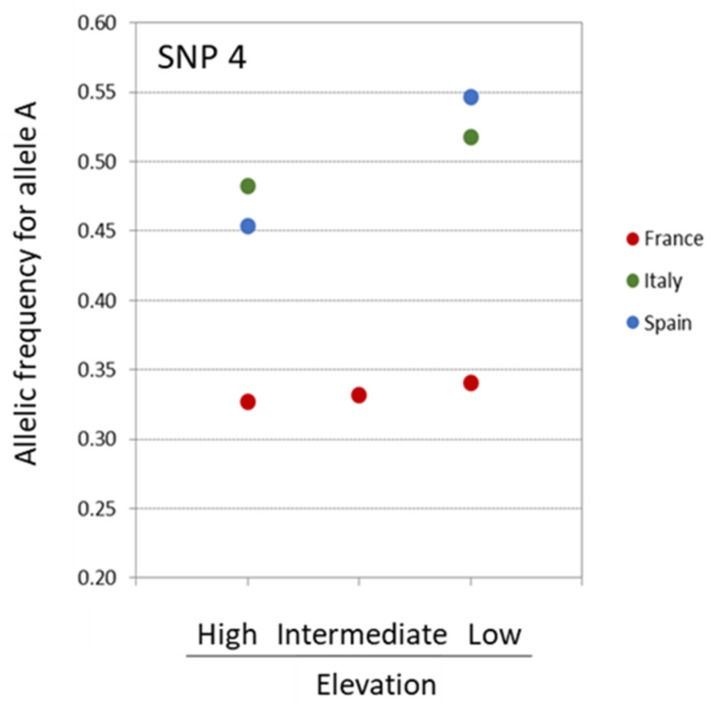
Distribution of the frequency of allele A for SNP 4 detected under selection by PCAdapt, Bayenv2, and Baypass.

Two of the outlier SNPs potentially targeted by selection, SNP 4 and SNP 378, were remarkable in that they had allelic frequency patterns compatible with adaptation along the studied altitudinal gradients. The allelic frequency distribution of those top-candidate SNPs revealed several patterns: the allelic frequency distribution of SNP 4 indicated that allele A was at higher frequency in low elevation sites across the three countries, while its frequency was intermediate for the French site located at intermediate elevation (Figure 3).

**Figure 4:**
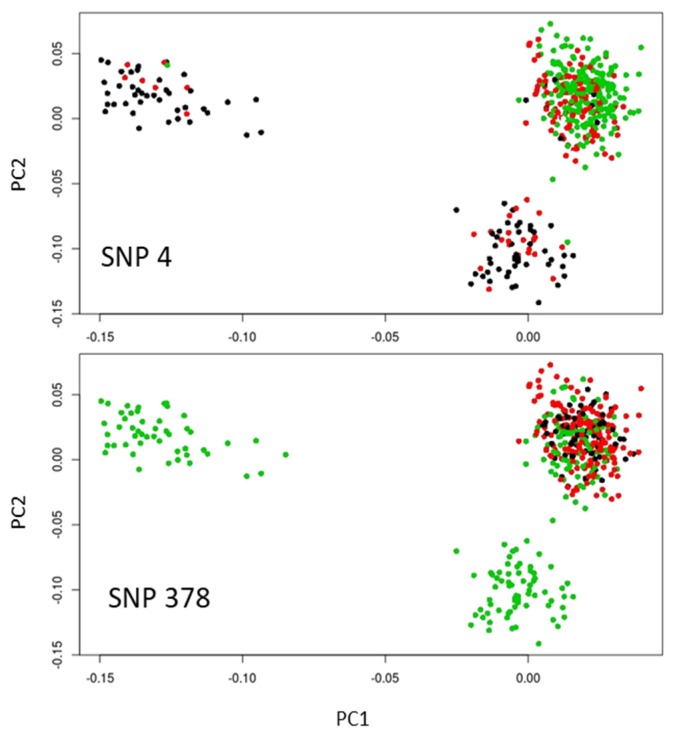
Highlights on the score plot for the two main SNP outliers common to the outlier methods based on the *XtX* statistics (Bayenv2 and Baypass) and PCA (PCAdapt). Homozygotes for allele 1 and 2 are in green and black, respectively, while heterozygotes are in red. The populations are the same as in Figure 1c.

The allelic frequency distribution was somewhat similar for SNP 378 with the highest proportion of allele A associated with the site of highest altitude, while this proportion was lower for the intermediate and lowest altitude sites. However, this pattern was true only for the French replicates, with the Italian and Spanish replicates being monomorphic for the considered allele (Figure 4). Thus, only SNP 4 showed a pattern of allelic frequency across the three sets of populations compatible with convergent evolution under similar selection pressure linked to elevational gradient.

## 4. Discussion

Population structure analyses suggest that the Italian populations experienced a distinct evolutionary history when compared to the French and Spanish populations. This result is in agreement with the main genetic clusters previously defined: French and Spanish populations belong to the Western cluster of Aleppo pine and display lower genetic diversity when compared to the Italian populations that are part of the Eastern/Southern cluster [20]. Therefore, our sampling covered not only contrasting environmental conditions within population replicates (i.e., altitudinal gradient), but also distinct evolutionary genetic units. This sampling design allowed testing whether the pattern of genetic divergence among heterogeneous environments held between different genetic clusters. Sampling design (i.e., both sampling strategy and location) is one of the most influential factors when performing outlier detection tests in studies with simulated data [2,39]. Only recently have empirical analyses integrated an experimental approach based on replicated population pairs to increase the power of outlier detection [9,10]. 

Discovering genes potentially targeted by natural selection also relies on statistical frameworks that differ in the way they summarize complex data, and therefore could indicate different loci under selection. This outcome has been seen in both theoretical [39,57] and empirical [12,60,61] works including the present study. As of yet, there is no single widely accepted approach on how to combine statistical tests from multiple genome scan methods [40,49]. Even if the model specification is correct and the tests are properly calibrated, taking the intersection of the set of outlier SNPs leads to the identification of too few significant outliers, while taking the union leads to too many. On one hand, combining several well-calibrated tests can decrease the sensitivity to particular models and lead to more robust testing approaches [49]. On the other hand, by taking the intersection of SNPs, even if some have extreme *p*-values under some tests, we assume that the discarded loci are false-positives because the underlying assumptions of the respective tests are wrong. Finally, this conservative approach may lead to overlooking loci under weak selection. There are some indications that such SNPs exist in conifers [61]: local adaptation could result mainly from small, potentially undetectable, covarying shifts in frequency at many loci [2]. Looking for polygenic adaptation adds an extra layer of complexity when trying to disentangle the effects of demographic history and selection, and several methods have been recently developed to address this issue [62,63,64,65,66]. 

Locus annotation could be obtained only for one of the two top-candidate SNPs, SNP 4, which is located in a non-coding region of a PIN-like protein (PIN2 in *Pinus tabuliformis*; GenBank: AJP06341.1). This family of proteins act as auxin transporters and constitute key regulators in multiple developmental events ranging from embryogenesis through morphogenesis and organogenesis to growth responses to environmental stimuli [67]. The other top-candidate, SNP 378, which was detected by the two Bayesian linear models in association with annual precipitation in the French populations, was also detected independently using the Bayenv2 linear model in relation to the precipitation of the driest month in a study on the whole distribution range of Aleppo pine [20]. Altogether, these results point to SNP 378 as a very good candidate for selection and indicate that precipitation is a potential predominant driver of selection at two distinct spatial scales. The relevance of the precipitation regime for Aleppo pine has been emphasized in various disciplines such as ecophysiology [26,28,68], species distribution modeling [25], population genetics [20,27] and quantitative genetics [29]. 

The allelic frequency distribution of the two top-candidate SNPs highlights the importance of taking into account several aspects when interpreting the outputs of selection tests at a local scale: (i) the evolutionary history of the species (previous studies showed that Aleppo pine western populations are genetically depleted, [e.g. 20], which constitutes a limitation to detect polymorphic markers in the Spanish and French populations); (ii) the sampling size in terms of individuals per population (much more precise allelic frequencies could be obtained for the French population composed of 356 individuals compared to the Italian and Spanish populations comprising 50 and 70 individuals, respectively); and (iii) the sampling size in terms of replicated environmental gradients (three sites are somewhat limited to draw general conclusions, especially if some populations are monomorphic for the targeted SNPs). When detected, a convergent pattern of microevolution at local geographical scales may be attributed to parallel selective pressures at the locus, shared ancestral standing variation, or spread of the selected alleles via gene flow. Understanding the origin of convergent evolution can assist in dissecting the basis of local adaptation, and thereby in predicting adaptive response to selection [69,70]. In Aleppo pine, the selective pressure along an elevational cline could allow the species to cope with current and future climate changes characterized by higher temperature and lower precipitation in the Mediterranean Basin (International Panel on Climate Change, Fifth Assessment Report [71]). Moving uphill would allow individuals to be exposed to higher precipitation levels without latitudinal or longitudinal migration [72,73].

## 5. Conclusions

With the combination of the approaches used here (selection of putative candidate genes for genotyping, paired sampling technique, and multiple statistical approaches), eight SNPs were identified as outliers by at least two methods, of which only two (SNP 4 and SNP 378) had their allelic frequencies associated with elevational gradients, and one (SNP 4) across all population replicates. Looking for signal of convergent evolution at local or regional scales is an attractive and powerful approach to understand the molecular basis of adaptation. However, false positives could be linked not only to the statistical methods used, but also to the sampling. The outcome of our study cautions against considering outlier markers detected by genomic scans as automatic candidates for convergent adaptation in populations submitted to similar selective pressures.

## Figures and Tables

**Table 1:** Summary statistics of the 14 outlier loci identified using the three outlier methods of the study.

**Bayenv2**	**SNP**	**Sequence**	***XtX* Value**
	149	seq-8671-529	20.42
	4	seq-9882-801	20.83
	316	seq-10373-2483	21.52
	378	seq-2_3941_01-381	23.76
Baypass	SNP	Sequence	*XtX* value
	378	seq-2_3941_01-381	13.44
	149	seq-8671-529	14.14
PCAdapt	SNP	Sequence	*p*-value
	335	seq-1_6493_01-100	2.64E-009
	94	seq-55383-900	1.74E-007
	19	seq-55383-1485	2.05E-007
	331	seq-55383-141	2.95E-007
	258	seq-9882-2209	4.74E-004
	4	seq-9882-801	7.23E-004
	384	seq-0_3073_01-92	1.21E-003
	281	seq-16094-410	3.36E-003
	10	seq-44358-1615	3.51E-003
	113	seq-44358-2515	3.80E-003
	269	seq-16094-1379	5.90E-003

**Table 2:** SNPs that coincided in being outliers for the same environmental variables (Env.) for both Bayesian linear models performed with Bayenv 2 and Baypass.

SNP	Sequence	Env.	BF Bayenv2	eBPis Baypass
169	seq-0_10162_01-244	BIO9	41.97	5.48
316	seq-10373-2483	Elevation	20.90	3.77
378	seq-2_3941_01-381	BIO12	47.38	3.65

BIO 9: Mean temperature of driest quarter; BIO 12: Annual precipitation.

**Table 3:** Annotation of the six loci where eight SNPs were found under selection with at least two methods.

SNP	Sequence	Description	*E*-Value
4, 258	seq-9882	PIN-like protein in various conifers	1e−96
149	seq-8671	No significant similarity found	
169	seq-0_10162_01	Anonymous locus in *Pinus taeda*	1e−86
269, 281	seq-16094	Anonymous locus in *Picea glauca*	1e−51
316	seq-10373	Putative alpha-xylosidase (XYL1) in *Pinus pinaster*	2e−99
378	seq-2_3941_01	Anonymous locus in *Pinus taeda*	5e−91

**Table 4:** SNPs found under selection by both PCAdapt and Baypass using the linear model to find SNPs associated with different environmental variables (Env.).

SNP	Contig	*p*-Value PCAdapt	eBPis Baypass	Env.
4	seq-9882-801	7.23E−004	5.38	BIO12
258	seq-9882-2209	4.74E−004	5.06	Elevation
258	seq-9882-2209	4.74E−004	6.34	BIO12
258	seq-9882-2209	4.74E−004	3.02	BIO19
269	seq-16094-1379	5.90E−003	4.86	BIO12
269	seq-16094-1379	5.90E−003	3.77	BIO16
269	seq-16094-1379	5.90E−003	4.72	BIO19
281	seq-16094-410	3.36E−003	3.21	Elevation
281	seq-16094-410	3.36E−003	5.21	BIO11
281	seq-16094-410	3.36E−003	3.03	BIO12
281	seq-16094-410	3.36E−003	4.64	BIO16
281	seq-16094-410	3.36E−003	4.92	BIO19

BIO 11: Mean temperature of coldest quarter; BIO 12: Annual precipitation; BIO 16: Precipitation of wettest quarter; BIO 19: Precipitation coldest quarter.

## Supplementary Materials

The following are available online at https://www.mdpi.com/2073-4425/10/9/673/s1, Figure S1: Likelihood of K for each value of K estimated in STRUCTURE; Figure S2: Scree plot that displays the percentage of variance explained by each PC; Figure S3: Distribution of the empirical p-values obtained by PCAdapt visualized through a Manhattan plot and a QQ-plot; Figure S4: Heatmaps of the pairwise *F*_ST_ distance and the covariance matrix calculated in Bayenv2; Figure S5: The *XtX*s estimated from Bayenv2 and from Baypass; Figure S6: Correlation matrix comparing the $\hat{\Omega }$ values amongst the populations of the SNP dataset computed in Baypass; Figure S7: Correlation matrix visualized as a hierarchical cluster tree where the relationships between populations can be appreciated compared to a neighbor joining tree of pairwise *F*_ST_; Table S1: Sampling details of the seven populations; Table S2: Environmental variables retrieved from WORDLCLIM for the seven populations; Table S3: List of bioclimatic variables BIO1–BIO19 available in WORDLCLIM; Table S4: Pairwise *F*_ST_ between the seven populations from France, Italy, and Spain; Table S5: Summary of the results of the Bayesian linear model performed in Bayenv2 with different bioclimatic variables; Table S6: Summary of the results of the Bayesian linear model performed in Baypass with different bioclimatic variables.
