# Supplementary material for: Looking for Local Adaptation: Convergent Microevolution in Aleppo Pine (Pinus halepensis)
Source: Genes (Basel). 2019 Sep 4;10(9):673. doi: 10.3390/genes10090673 (PMC6771008; doi:10.3390/genes10090673)
Supplement: Supplementary file 1 [file genes-10-00673-s001.pdf]

Supporting Information

Table S1: sampling details of the seven populations.

Country	Plot	Coordinates		Sampling size
		Latitude	Longitude	
France	Saint Mitre	43.4518140	5.0419330	50
France	Font Blanche	43.2407610	5.6791030	256
France	Siou Blanc	43.2375560	5.8875000	50
Italy	Mattinata	41.6949892	16.0589908	25
Italy	Monte Sant' Angelo	41.6949104	16.0216220	25
Spain	Alzira	39.1223287	-0.3892886	39
Spain	Montan	40.0472534	-0.5925626	31

Table S2: environmental variables retrieved from WORDLCLIM for the seven populations.

Population	altitude	BIO 1	BIO 2	BIO 3	BIO 4	BIO 5	BIO 6	BIO 7	BIO 8	BIO 9	BIO 10	BIO 11	BIO 12	BIO 13	BIO 14	BIO 15	BIO 16	BIO 17	BIO 18	BIO 19
Font Blanche	408	126	83	34	5505	257	17	240	97	198	198	58	711	90	17	33	253	94	94	206
Siou Blanc	614	116	80	34	5419	245	10	235	89	187	187	50	777	94	20	32	273	107	107	226
St Mitre	138	134	96	36	5885	279	13	266	142	210	210	59	614	85	18	33	220	89	89	164
Monte Sant' Angelo	462	135	69	29	5958	265	33	232	108	214	214	65	535	63	29	25	180	93	93	156
Mattinata	79	160	76	32	5783	289	55	234	135	235	235	90	465	60	20	35	173	69	69	143
Montan	848	123	94	36	5878	265	10	255	132	54	202	54	490	59	23	29	158	93	106	93
Alzira	112	173	97	40	5197	301	60	241	187	239	243	109	466	81	9	48	191	50	80	120

Table S3: list of bioclimatic variables BIO1-BIO19 available in WORDLCLIM.

Name	Description
BIO1	Annual Mean Temperature
BIO2	Mean Diurnal Range (Mean of monthly (max temp - min temp))
BIO3	Isothermality (BIO2/BIO7) (* 100)
BIO4	Temperature Seasonality (standard deviation *100)
BIO5	Max Temperature of Warmest Month
BIO6	Min Temperature of Coldest Month
BIO7	Temperature Annual Range (BIO5-BIO6)
BIO8	Mean Temperature of Wettest Quarter
BIO9	Mean Temperature of Driest Quarter
BIO10	Mean Temperature of Warmest Quarter
BIO11	Mean Temperature of Coldest Quarter
BIO12	Annual Precipitation
BIO13	Precipitation of Wettest Month
BIO14	Precipitation of Driest Month
BIO15	Precipitation Seasonality (Coefficient of Variation)
BIO16	Precipitation of Wettest Quarter
BIO17	Precipitation of Driest Quarter
BIO18	Precipitation of Warmest Quarter
BIO19	Precipitation of Coldest Quarter

Table S4: pairwise F_{ST} between the seven populations from France, Italy and Spain. Significant difference (p-val <0.5) among population pairs are indicated with an asterisk.

	France			Spain		Italy	
	Font Blanche	Siou Blanc	Saint Mitre	Montan	Alzira	Monte Sant' Angelo	Mattinata
Font Blanche	0	0.0121*	0.0049*	0.0853*	0.0999*	0.3082*	0.3073*
Siou Blanc		0	0.0138*	0.1056*	0.1210*	0.3124*	0.3115*
Saint Mitre			0	0.0861*	0.0990*	0.3254*	0.3232*
Montan				0	0.0151*	0.2978*	0.2877*
Alzira					0	0.2760*	0.2663*
Monte Sant' Angelo						0	0.0122
Mattinata							0

Table S5: summary of the results of the Bayesian linear model performed in Bayenv2 with different bioclimatic variables.

**SNP**	**Sequence**	**Bioclimatic variable**<br>**BF**		
169	seq-0_10162_01-244	altitude	BIO9	
		20.903	41.971	
312	seq-UMN_3408_01-293	BIO2		
		20.853		
316	seq-10373-2483	BIO2	BIO19	
		20.505	53.894	
378	seq-2_3941_01-381	BIO12	BIO16	BIO19
		47.378	64.627	71.261

Table S6: summary of the results of the Bayesian linear model performed in Baypass with different bioclimatic variables (Env.).

SNP	Sequence	Env	M-pearson	SD_Pearson	eBPis
2	seq-6890-2409	BIO3	-0.5733702	0.23280148	3.11399102
4	seq-9882-801	BIO12	-0.5251087	0.1238397	5.37596943
7	seq-7270-1484	BIO2	-0.8051462	0.11801382	4.14633727
7	seq-7270-1484	BIO7	-0.7214707	0.1499661	3.06285797
7	seq-7270-1484	BIO12	0.76639455	0.08623712	4.46058182
7	seq-7270-1484	BIO16	0.72976022	0.13401115	3.03150769
7	seq-7270-1484	BIO19	0.7887976	0.11145945	3.75236008
30	seq-9243-371	BIO12	0.64362547	0.14199203	5.24558596
30	seq-9243-371	BIO16	0.59596188	0.1386626	3.71530188
30	seq-9243-371	BIO19	0.58877171	0.14257403	3.97485605
148	seq-0_12216_02-537	BIO12	-0.7620282	0.15270792	4.15829304
148	seq-0_12216_02-537	BIO19	-0.7102334	0.16325765	3.07555576
151	seq-0_8992_01-119	BIO12	0.65511587	0.19022025	3.07242975
169	seq-0_10162_01-244	Altitude	0.76153998	0.13068642	3.76859495
169	seq-0_10162_01-244	BIO1	-0.6542224	0.14417419	3.09435782
169	seq-0_10162_01-244	BIO9	-0.8379422	0.08956155	5.48337617
169	seq-0_10162_01-244	BIO11	-0.6239025	0.14530298	3.00001402
169	seq-0_10162_01-244	BIO13	-0.7137226	0.12101459	5.19887839
169	seq-0_10162_01-244	BIO15	-0.6434817	0.15061643	3.08844092
182	seq-0_16860_01-314	Dry/Wet	0.8022037	0.14918111	3.00958542
205	seq-CL708CONTIG1_02-173	BIO12	-0.762647	0.17323487	3.24830529
258	seq-9882-2209	Altitude	0.47978421	0.14401977	5.0545209
258	seq-9882-2209	BIO12	-0.5801209	0.11965805	6.33377503
258	seq-9882-2209	BIO19	-0.4719654	0.14022928	3.01268994
269	seq-16094-1379	BIO12	0.6201313	0.13851844	4.85579828
269	seq-16094-1379	BIO16	0.63737081	0.13333415	3.77450901
269	seq-16094-1379	BIO19	0.67220926	0.12747789	4.72441191

281	seq-16094-410	Altitude	-0.447868	0.16543734	3.2116434
281	seq-16094-410	BIO12	0.58038238	0.135169	5.20973055
281	seq-16094-410	BIO12	0.57368845	0.13367159	3.02821708
281	seq-16094-410	BIO16	0.64456484	0.12215464	4.64057127
281	seq-16094-410	BIO19	0.63426007	0.12530935	4.91602902
316	seq-10373-2483	Altitude	-0.5209439	0.13402176	4.36836379
316	seq-10373-2483	BIO12	-0.525043	0.13970482	3.71427816
316	seq-10373-2483	BIO12	0.67126778	0.10078796	6.71104438
316	seq-10373-2483	BIO16	0.58991637	0.1342952	3.57113029
316	seq-10373-2483	BIO19	0.67505682	0.1205309	5.17564163
325	seq-8188-285	Altitude	-0.5498621	0.2263624	3.03248511
325	seq-8188-285	BIO12	0.75402557	0.16680693	3.51109846
337	seq-36858-735	BIO10	0.56181138	0.15827186	3.08795106
337	seq-36858-735	BIO12	-0.6011001	0.17039471	3.3155499
364	seq-2_2937_01-309	BIO2	-0.8005791	0.12499065	4.36356318
364	seq-2_2937_01-309	BIO7	-0.6979643	0.15040826	3.26117794
364	seq-2_2937_01-309	BIO12	0.76465655	0.0946094	4.85192671
364	seq-2_2937_01-309	BIO16	0.70987328	0.14077677	3.14422469
364	seq-2_2937_01-309	BIO19	0.76967958	0.11920607	4.04043984
378	seq-2_3941_01-381	BIO12	0.51160742	0.15811359	3.65435041

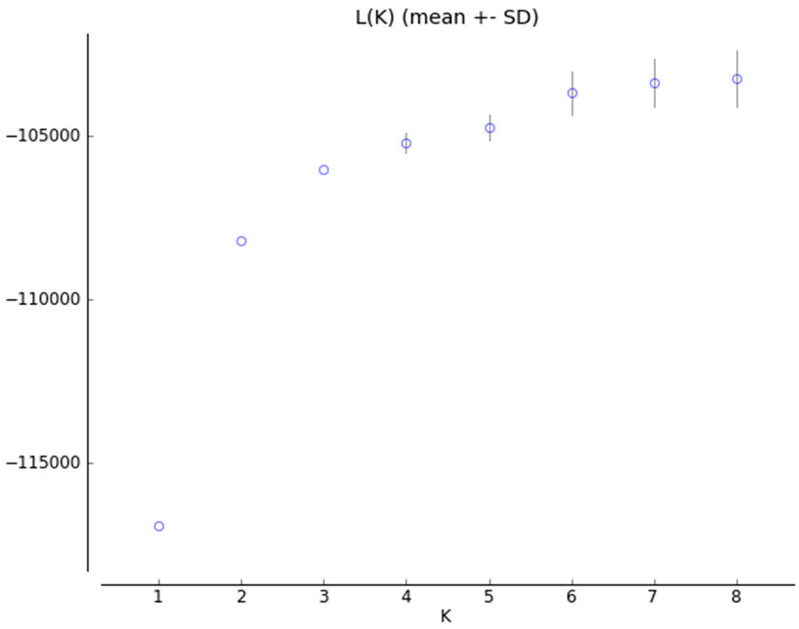

Figure S1: Likelihood of *K* (averaged across all six iterations) for each value of *K* (1 to 8) estimated in STRUCTURE.

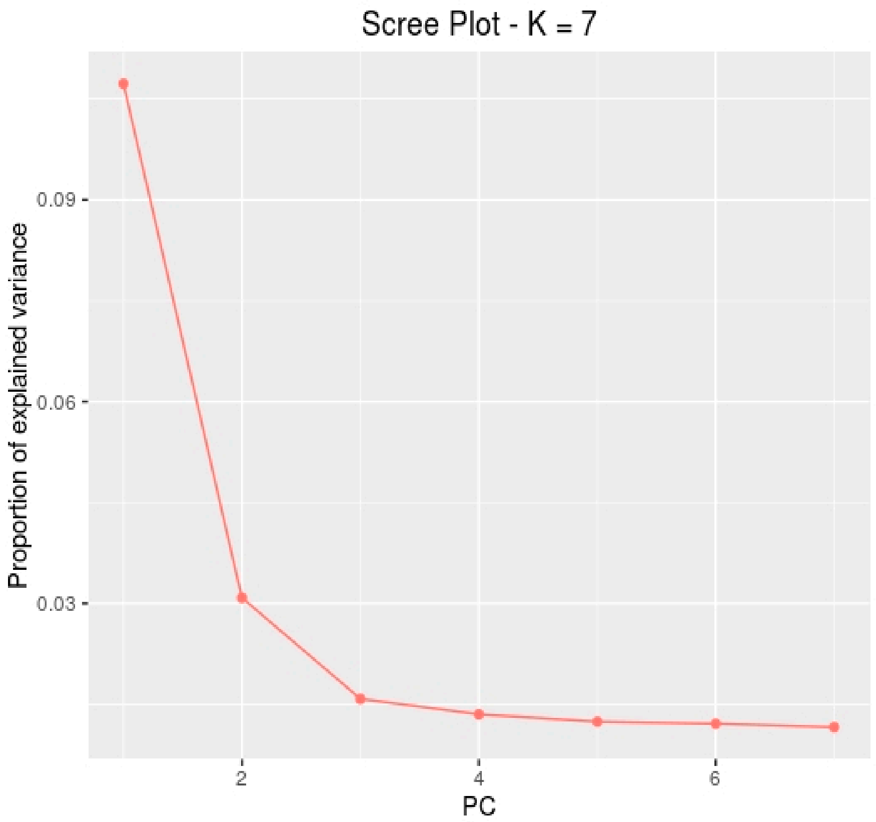

Figure S2: Scree plot that displays, in decreasing order, the percentage of variance explained by each PC. These correspond to the eigenvalues in decreasing order.

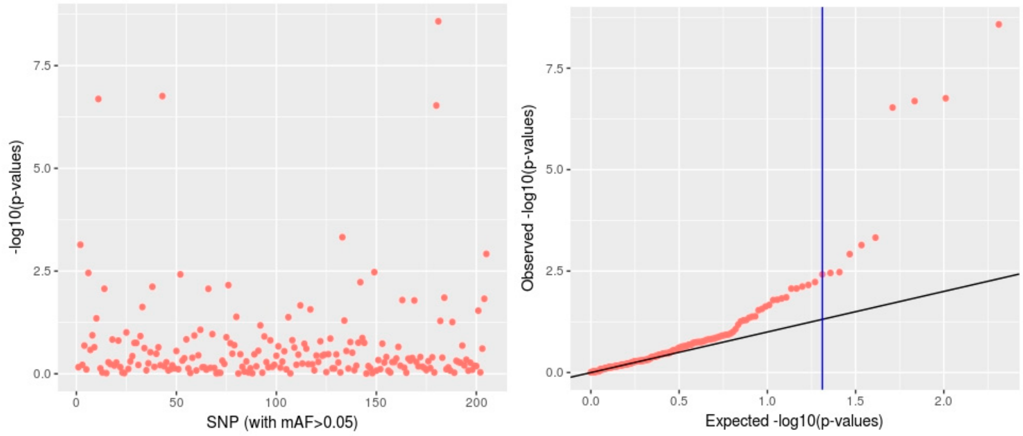

Figure S3: Distribution of the empirical p-values obtained by PCAdapt visualized through a Manhattan plot (left) and a QQ-plot (right) showing the cut off of 0.1% (vertical blue line).

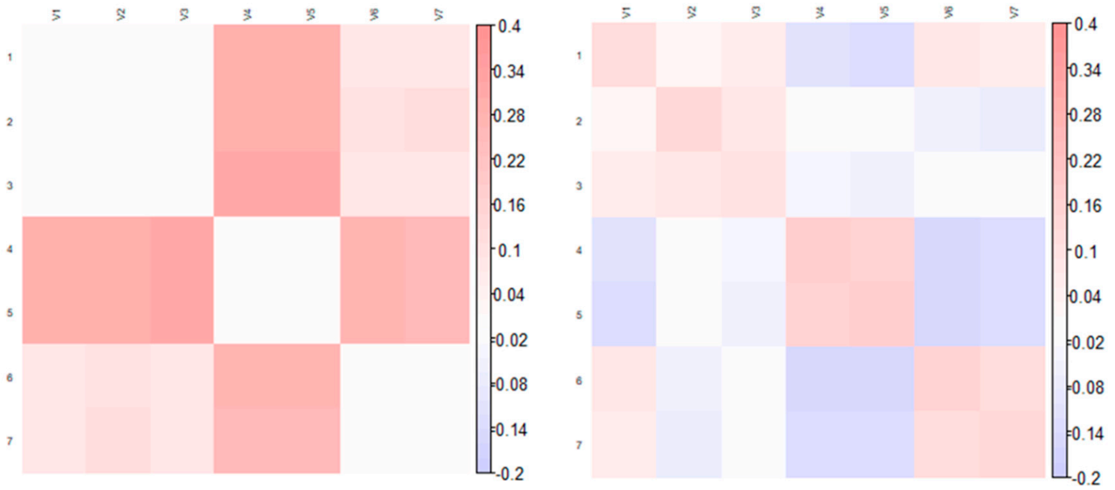

Figure S4: Heatmaps of the pairwise F_{ST} distance (left) and the covariance matrix calculated in Bayenv2 (right). Number corresponds to the following populations; 1: Font Blanche (France), 2: Siou Blanc (France), 3: Saint Mitre (France), 4: Monte Sant' Angelo (Italy), 5: Mattinata (Italy), 6: Montan (Spain) and 7: Alzira (Spain).

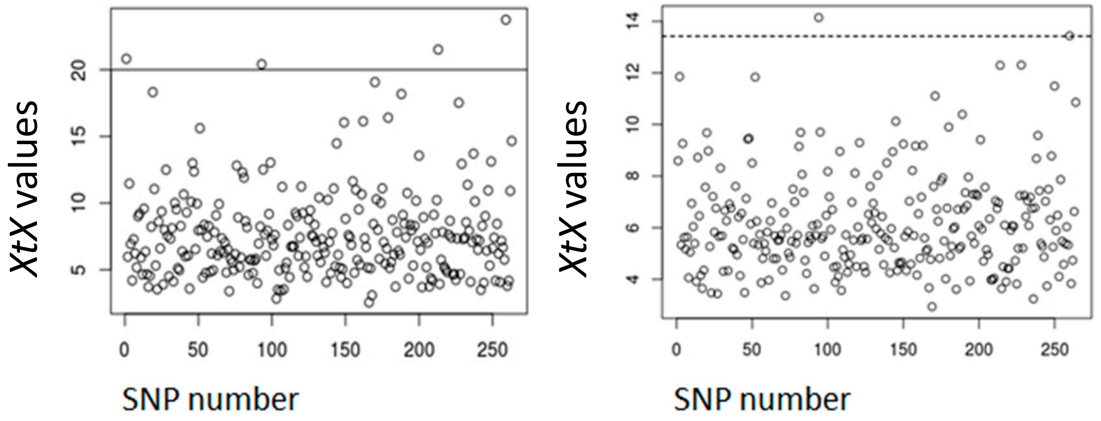

Figure S5: The *XtX*s estimated from Bayenv2 (left) with a threshold based on a cut off from the distribution (horizontal dark line) and from Baypass (right) showing the 0.01 per cent cut off threshold (horizontal dotted line) computed using the POD data.

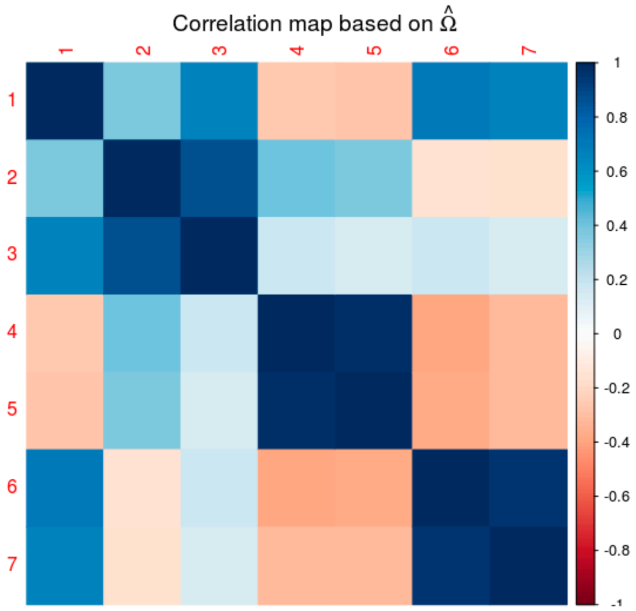

Figure S6: A correlation matrix comparing the $\widehat{\Omega}$ values amongst the populations of the SNP data set computed in Baypass. Numbers correspond to the following populations; 1: Font Blanche (France), 2: Siou Blanc (France), 3: Saint Mitre (France), 4: Monte Sant' Angelo (Italy), 5: Mattinata (Italy), 6: Montan (Spain) and 7: Alzira (Spain).

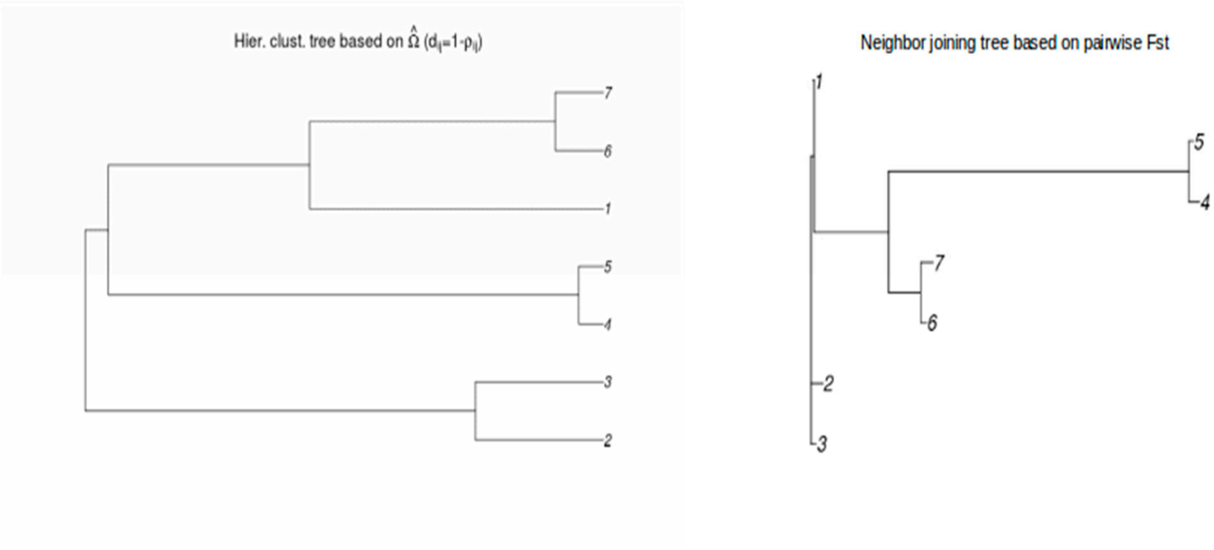

Figure S7: The correlation matrix visualised as a hierarchical cluster tree where the relationships between populations can be appreciated (left) compared to a neighbour joining (right) tree of pairwise F_{ST}. Number corresponds to the following populations; 1: Font Blanche (France), 2: Siou Blanc (France), 3: Saint Mitre (France), 4: Monte Sant' Angelo (Italy), 5: Mattinata (Italy), 6: Montan (Spain) and 7: Alzira (Spain).
